# Screening of hub genes for sepsis-induced myopathy by weighted gene co-expression network analysis and protein-protein interaction network construction

**DOI:** 10.1186/s12891-024-07967-0

**Published:** 2024-10-22

**Authors:** Jianhao Wang, Kun Han, Jinshuai Lu

**Affiliations:** 1 Postgraduate School, Xinjiang Medical University, Xinjiang, 830000 China; 2 Department of Emergency, People’s Hospital of Xinjiang Uygur Autonomous Region, No 91, Tian Chi Road, Xinjiang, 830001 China

**Keywords:** Sepsis, Sepsis-induced myopathy (SIM), Weighted gene co-expression network analysis (WGCNA), Protein-protein interaction network (PPI), Gene set enrichment analysis (GSEA)

## Abstract

Sepsis-induced myopathy is one of the serious complications of sepsis, which severely affects the respiratory and peripheral motor systems of patients, reduces their quality of life, and jeopardizes their lives, as evidenced by muscle atrophy, loss of strength, and impaired regeneration after injury. The pathogenesis of sepsis-induced myopathy is complex, mainly including cytokine action, enhances free radical production in muscle, increases muscle protein hydrolysis, and decreases skeletal muscle protein synthesis, etc. The above mechanisms have been demonstrated in existing studies. However, it is still unclear how the overall pattern of gene co-expression affects the pathological process of sepsis-induced myopathy. Therefore, we intend to identify hub genes and signaling pathways. Weighted gene co-expression network analysis was our main approach to study gene expression profiles: skeletal muscle transcriptome in ICU patients with sepsis-induced multi-organ failure (GSE13205). After data pre-processing, about 15,181 genes were used to identify 13 co-expression modules. Then, 16 genes (FEM1B, KLHDC3, GPX3, NIFK, GNL2, EBNA1BP2, PES1, FBP2, PFKP, BYSL, HEATR1, WDR75, TBL3, and WDR43) were selected as the hub genes including 3 up-regulated genes and 13 down-regulated genes. Then, Gene Set Enrichment Analysis was performed to show that the hub genes were closely associated with skeletal muscle dysfunction, necrotic and apoptotic skeletal myoblasts, and apoptosis in sepsis-induced myopathy. Overall, 16 candidate biomarkers were certified as reliable features for more in-depth exploration of sepsis-induced myopathy in basic and clinical studies.

**Supplementary Information:**

The online version contains supplementary material available at 10.1186/s12891-024-07967-0.

## Introduction

Sepsis is currently defined as life-threatening organ dysfunction resulting from a dysregulated host response to infection [1]. Sepsis and septic shock continue to be the leading cause of death globally, affecting the health of millions of people each year and causing between one-third and one-sixth of all deaths [2–4]. Sepsis has emerged as one of the major challenges in the field of critical care, and early diagnosis as well as aggressive treatment can improve in-hospital mortality and overall survival after sepsis [5, 6]. The skeletal muscular system is one of the major organ systems damaged in sepsis patients. The resulting neuromuscular dysfunction and impaired regenerative capacity are known as sepsis-induced myopathy (SIM) and is characterized by muscle atrophy, loss of strength, and impaired regeneration after injury [7]. SIM is one of the severe complications of sepsis, which seriously affects the respiratory system and peripheral motor system of patients, reduces the life quality of patients, and jeopardizes life safety. SIM is a major cause of mortality and long-term morbidity in critically ill patients, but the incidence and prevalence of SIM is not known. The mechanisms of SIM are complex, and processes now identified as playing a pivotal role in regulating sepsis-induced muscle damage include: (1) Effects of cytokines: In the setting of sepsis and systemic inflammatory response syndrome, various cytokines and chemokines are markedly elevated in vivo, and current research shows that prolonged exposure to cytokines in the in vitro environment of muscle tissue or muscle cells isolated from the body decreases muscle specific force but does not isolate muscle bundles or alter muscle mass or protein levels [8, 9]. However, prolonged exposure of cultured myoblasts to cytokines decreases cell size and protein content [10]. (2) Sepsis enhances the production of free radicals in muscle: Cytokines are markedly elevated during the pathology of sepsis, which leads to the overproduction of free radicals in several organs, including skeletal muscle. Simultaneously, many excellent reviews emphasize the importance of free radicals in redox regulation and pathogenic dysregulation of skeletal muscle performance in several conditions [11–13]. Many studies exist to prove that excess free radical production plays a crucial role in SIM. (3) Sepsis increases muscle protein hydrolysis: it has been shown that several components of the proteasome protein hydrolysis degradation system are upregulated in skeletal muscle in both infected patients and animal models of systemic inflammation [14]. However, the proteasome cannot degrade intact muscle fiber proteins. Proteolysis in muscle requires an initial step of destroying the contractile matrix by activating calpain or cysteine asparaginase, followed by a second step of ubiquitination of the destroyed proteins and, finally, proteasome-mediated degradation [15]. Therefore, in the course of sepsis and systemic inflammatory response, it may be due to the activation of the above-mentioned muscle proteolytic process, which leads to muscle proteolysis, causing muscle atrophy and muscle contraction disorders, and, ultimately, sepsis-induced myopathy. (4) Sepsis decreases skeletal muscle protein synthesis: it is now well established that sepsis-induced myopathy is associated with a decrease in muscle mass, and in addition to focusing on the increased catabolism of muscle proteins, it is crucial to focus on the decrease in muscle protein synthesis that occurs during the pathological process of sepsis as well [16]. It has been shown that sepsis preferentially inhibits the synthesis of myofibrillar proteins and sarcoplasmic proteins in fast-twitch muscle and that overall muscle protein synthesis is reduced in severe sepsis, regardless of fiber type composition [17]. There are many current studies on the mechanisms and clinical features of SIM; however, the details of the co-expression of genes associated with the pathological process of SIM are unknown; therefore, elucidating potential genes associated with SIM may contribute to in-depth biological and clinical studies.

Weighted gene co-expression network analysis (WGCNA) is an efficient bioinformatics analysis method. Sets of genes with synergistic solid changes can be explored, and the strongest associated phenotypes can be verified. Consequently, the alteration technique is applied to various disciplines, especially in pathway and gene identification. WGCNA has also been used in the field of sepsis, successfully identifying the pathways associated with many of the potential genes. Proteins and their functional interactions form the backbone of cellular mechanisms. To fully understand biological phenomena, their connectivity networks need to be considered. Versatility, adaptability, and specificity of proteins allow them to create networks that control almost all intra- and intercellular activities. Protein-protein interaction (PPI) reveals the relative importance of proteins in different organisms and communities by analyzing these networks, which can improve our understanding of physiopathological mechanisms, help decipher gene-disease-drug associations, and find therapeutic approaches. In this study, we screened hub genes in SIM by constructing the WGCNA and PPI networks to provide new ideas for diagnosing and treating SIM.

## Methods

### Data acquisition and data handling

Data related to septic muscular dystrophy were obtained from GSE13205 (https://www.ncbi.nlm.nih.gov/geo/query/acc.cgiacc=GSE13205). They were used as a target set for the study for subsequent steps and to predict potential pivotal genes and factors associated with septic muscular dystrophy [18]. The dataset contains transcriptional profiling information of skeletal muscle tissues from sepsis patients in ICU and skeletal muscle tissues from normal control volunteers. The collected data were processed and analyzed using R. A set of DEGs with |logFC| ≥ 1 and *p* < 0.05 was obtained by using the Linear Models for Microarray Data (limma) tool in R to analyze the differences in the raw data [19]. After data preprocessing, the dataset from GSE13205 would be used for subsequent analysis as the sample.

### Protein-protein interaction (PPI) network construction

To ensure the best graphical display of DEGs protein interactions, the Search Tool for the Retrieval of Interacting Genes (STRING) database (http://string-db.org/) was used for generating a PPI network dataset [20]. The lowest required interaction score ≥ 0.9 was chosen as the cutoff value. Subclusters were created in the PPI network of septic muscular dystrophy using the Cytoscape software’s Molecular Complex Detection (MCODE) plugin. Advanced options were set to degree cutoff = 2; node score cut off = 0.2; K-Core = 2. Genes in subclusters with MCODE scores greater than 2 were used as candidate hub genes for subsequent analysis. The subclusters created in the PPI network were visualized using the R-package “igraph.”

### Construction of WGCNA

Networks were constructed for 15,181 genes obtained from the GSE13205 dataset using the R package “WGCNA” [21]. Scale independence and average connectivity were identified by network topology analysis at X-axis values in the interval 1–20. Meanwhile, a reasonable soft threshold was selected when the scale-free topology fitting index was greater than 0.9. In the gene dendrogram, based on the minimum module size set to 80 and applying hierarchical clustering, genes with similar expression characteristics are sorted into the same modules based on Topological Overlap Matrix (TOM) dissimilarity. The merge cut value is the height of the cuts made to the dendrogram during the module merging process and is related to the number and precision of the modules we finally collected. Finally, modules with highly homologous genes under dissimilarity less than the merge cut value are merged to obtain the key modules using the R package “flashclust” [22].

### Selection of key modules related to muscular dystrophy in Sepsis

The modules closely related to sepsis muscular dystrophy were obtained by hierarchical clustering and used as key modules for subsequent studies. Gene Significance (GS) indicates the gene level relative to the clinical phenotype. Module Significance (MS) indicates the average module significance of the genes associated with the entire module. Module Eigengene (ME) indicates the relationship between the whole module gene and the clinical phenotype, and the expression pattern of the module is described by calculating the relationship between the clinical phenotype and the module. Among them, the modules with the most significant MS and module-trait relationship were considered the most relevant and representative of septic muscular dystrophy.

### Functional enrichment analysis of key modules

To determine the specific role of genes in pathophysiological processes, the extracted key modules were analyzed by Gene Ontology (GO) and Kyoto Encyclopedia of Genes and Genomes (KEGG) using the R package “clusterProfile” [23].

### Screening candidate genes for hub genes

Using weighted co-expression networks, connectivity was estimated by the value of Pearson’s correlation analysis, which represents the sum of correlations between two genes for all intergenic connections. Intra-module connectivity was defined as the connectivity of individual genes in separate modules. Module membership (MM) indicates the level of a gene relative to the module eigengenes. First, the genes with GS > 0.2 and MM > 0.8 in the two modules exhibiting the highest positive and negative correlations were selected as the candidate hub genes. These candidates were then intersected with those identified differential genes and the PPI network. The overlapping genes from Venn diagrams through the R package “VennDiagram” would be used in the subsequent studies [24].

### Gene set enrichment analysis

Gene Set Enrichment Analysis (GSEA) was performed on individual hub genes to further screen the potential functions of hub genes involved in sepsis muscle atrophy. The expression levels of the DEGs were used as phenotypic labels, and the Pearson Correlation Coefficient was used as a measure of gene ordering. In this process, using the R package “clusterProfile,” the C2 and C5 gene collections of MSigDB (version 2024.1.Hs) were selected as the reference collection and the a priori gene collection for functional enrichment analysis [25]. The nominal p-value (NOM p-val) > 0.05 and the absolute value of the normalized enrichment score (NES) > 1, which was considered statistically significant. The enrichment results were visualized using the R package “enrichplot” [23].

### Receiver operating characteristic curve analysis

The receiver operating characteristic (ROC) curve, which meant assessments of the abilities of diagnostic tests for a biomarker, was achieved by the “pROC package” [26]. The test sensitivity was set at the y-axis, while the “1-specificity,” which referred to the false-positive rate, was set at the x-axis. Then, areas under the ROC curves were calculated to quantify ROC curves, and those values of areas were abbreviated to area under the curve (AUC). AUC could serve as an indicator of the ability to diagnose. The area’s value of AUC was supposed to be between 1.0 and 0.5. The closer the AUC was to 1.0, the more accurate the diagnosis would be.

### Analyses of statistics

It was R version 4.3.2 software that served as a tool to achieve all statistical processing and analysis. The significance of the statistical discrepancies was processed via a non-parametric method or t-test, depending on the data features. It was regarded as statistically significant when *p* < 0.05.

## Results

### Data acquisition and preprocessing

**Fig. 1:**
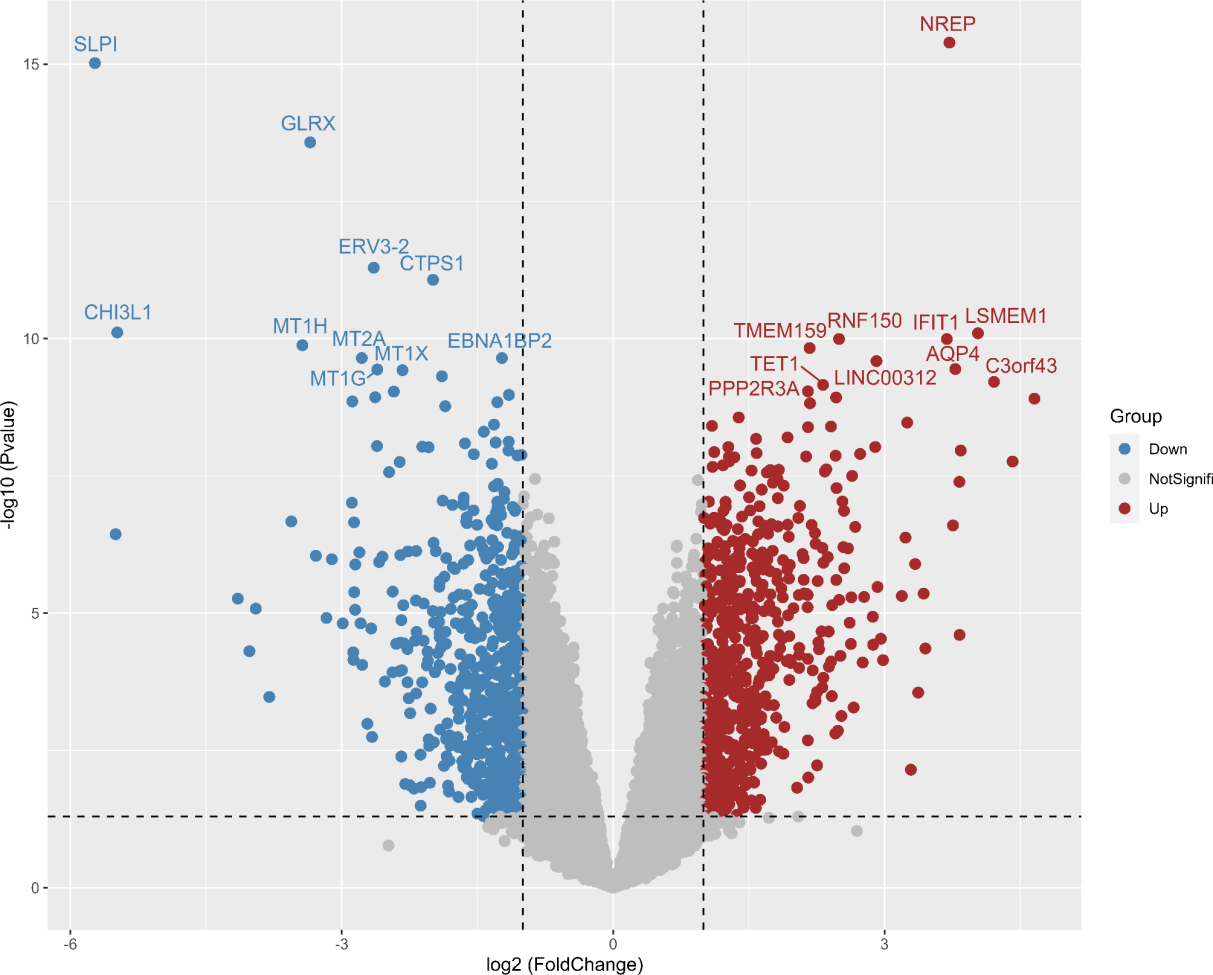
DEG analysis. Volcano plot of DEG analysis of GSE13205 dataset using limma package. The top 10 DEGs of the two groups have been marked

The GSE13205 dataset has 21 samples of microarray data, including 13 sepsis patients and 8 normal control volunteers, which use the gene chip platform GPL570. A total of 15,182 comparable gene expression profiles were obtained for subsequent analysis after data normalization for the same probe corresponding to multiple genes, different probes corresponding to the same gene, and the null value for correspondence processing. Using the limma package for DEG analysis, 1073 DEGs with |logFC|≥1, p, and adj.p.val less than 0.05 were obtained, including 566 up-regulated genes and 507 down-regulated genes (Fig. 1).

### PPI network construction

**Fig. 2:**
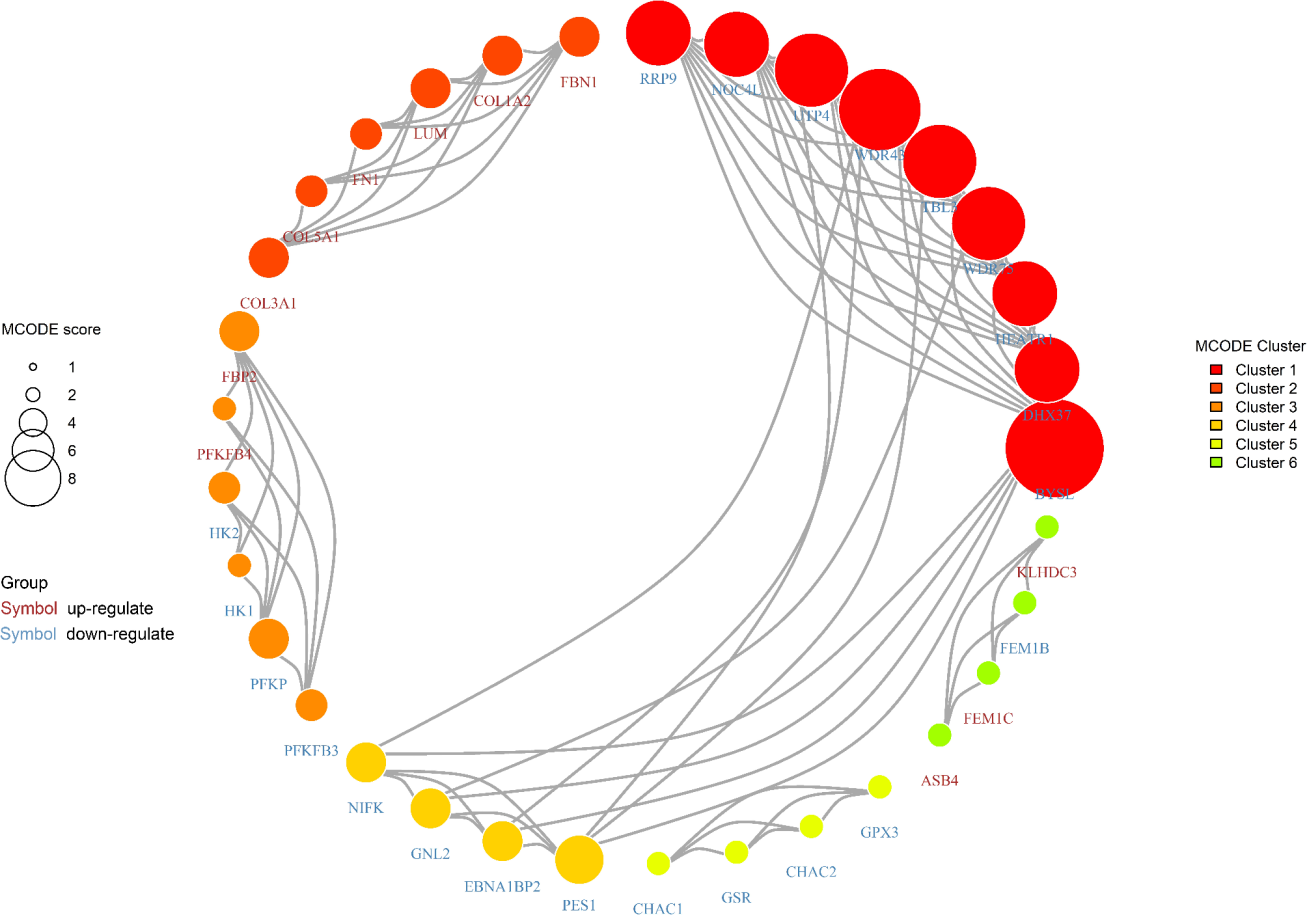
Subclusters of the PPI network were created using the Molecular Complex Detection Plugin (MCODE) in Cytoscape software. The subclusters with MCODE values > 2 were filtered out, in which each kind of Cluster1, Cluster2, Cluster3, Cluster4, Cluster5, and Cluster6 represents six subclusters, which are represented by different colors, with darker colors and larger volumes representing higher MCODE values. This figure shows that six subclusters contain 33 genes. The colors of the gene names in the figure represent the grouping of DEGs to which they belong, with brown representing the up-regulated group and blue representing the down-regulated group

The above DEGs were passed through the STRING database to construct the corresponding PPI networks, and the MCODE Plug-in in Cytoscape software was used to create sub-clusters for the PPI networks. A total of 21 subclusters were created, and the genes in which the MCODE values were > 2, i.e., the 6 subclusters shown in Fig. 2, with a total of 33, were used as hub genes for subsequent analysis.

### Screening hub gene modules for sepsis muscular dystrophy

**Fig. 3:**
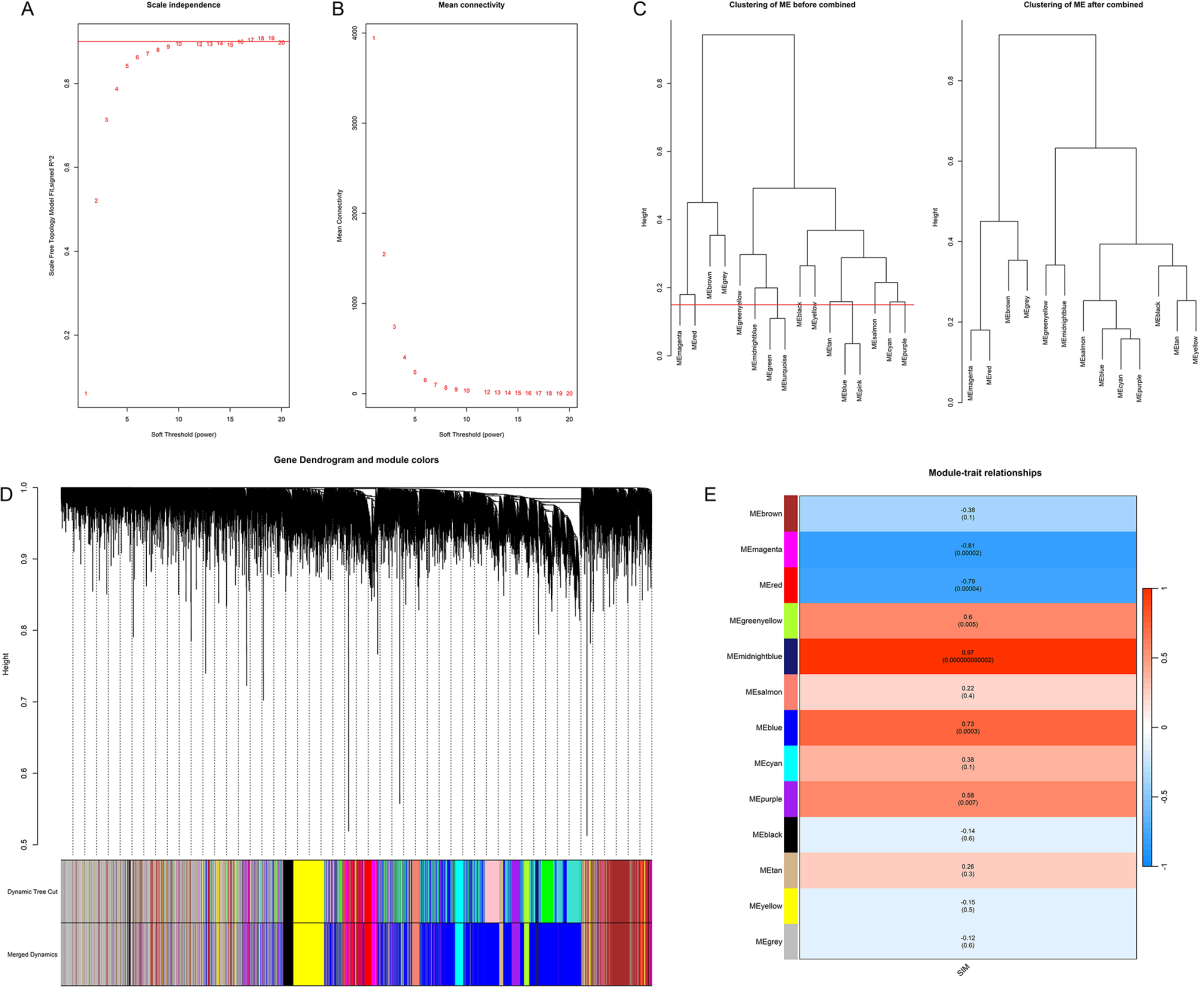
**A-E** Gene co-expression network. (**A**) Scale-free fitting parameters for different soft-threshold powers. (**B**) Average connectivity according to different soft threshold powers. (**C**) Module characterized gene clusters obtained by WGCNA. (**D**) Cluster dendrogram. Different colors represent the different co-expression modules we obtained, while black branches indicate the genes contained therein. (**E**) Heatmap showing individual modules connected to SIM. Correlation coefficients with p-values of ME-trait correlation and MS are included in the modules. The red color indicates a strong positive correlation between the module and SIM, while the blue color indicates a strong negative correlation

Sharp samples were identified by sample clustering, and the cutHeight parameter was set at 140 to exclude outlier samples (GSM333440). Network topology analysis was performed using the WGCNA package, and a soft threshold of 18 was selected through a built-in function. Modular enrichment analysis of genes was performed, the minimum module size was set to 80, and modules with differences less than 15% were merged using the flashcluster package. Green module and turquoise module, blue module and pink module were merged in pairs, and the dissimilarity between the merged blue and green was still less than 15%, so the combined blue module was merged again. Finally, 13 candidate modules were obtained. (Figs. 3A-E)

The two modules with the most positive and negative correlation with the phenotype were filtered based on the absolute value of Pearson correlation and the MS value of each module on each sample. Midnightblue module: contains 150 genes, cor: 0.97, p: 2.13E-12, MS: 0.76; and the magenta module: contains 525 genes, cor: -0.81, p: 1.85E-05, MS: 0.57 (Fig. 3E). However, considering the ME-trait correlation and the MS value of the red module is very close to the magenta module. Red module: containing 756 genes, cor: -0.79, p: 4.14e-05, MS: 0.56 (Fig. 3E). The genes in the above three modules were selected as candidate genes for the next validation step.

### Key module related functional analysis and screening of core molecules

**Fig. 4:**
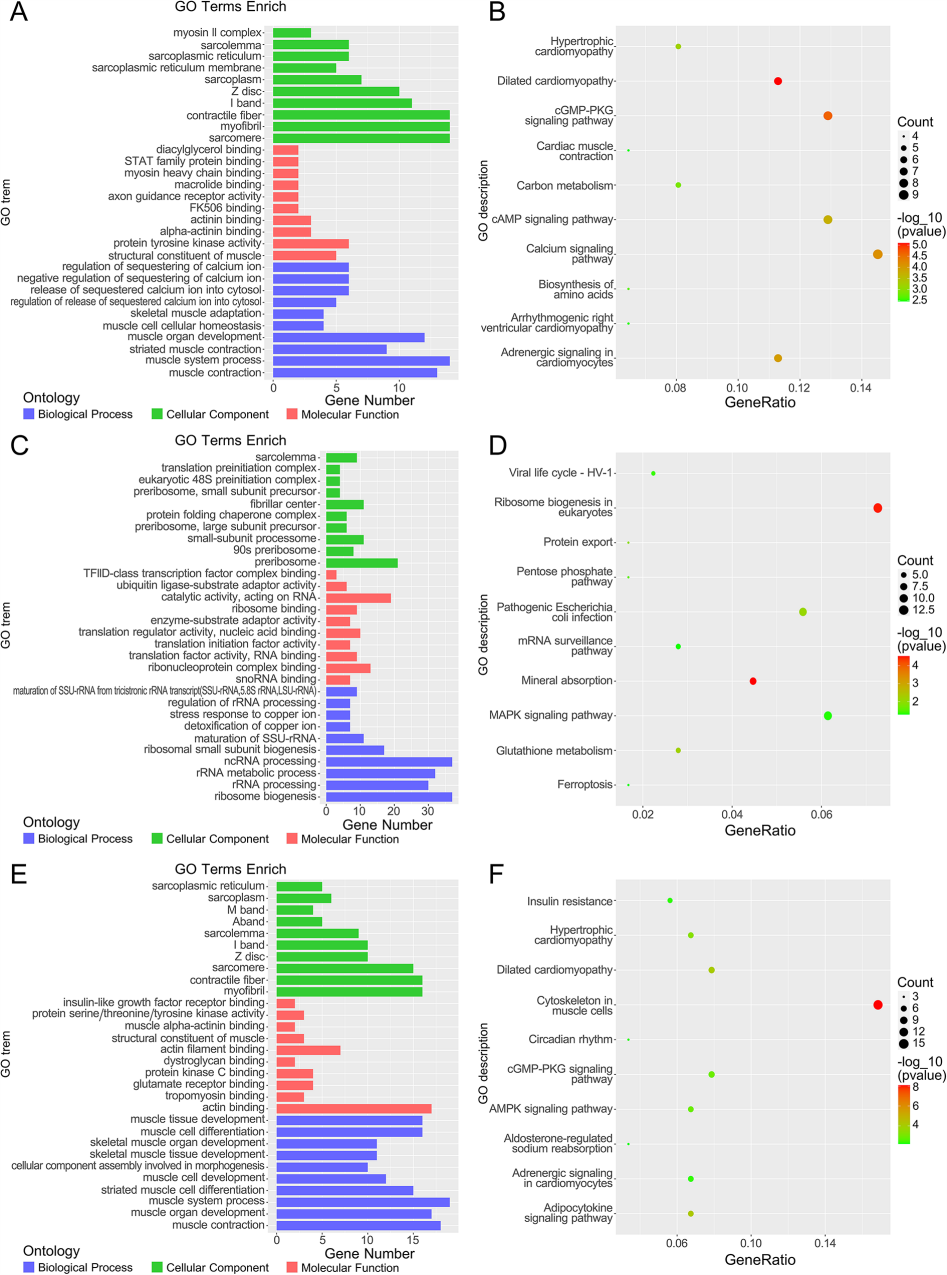
**A**-**F** GO and KEGG analysis of key modules. (**A**) GO analysis on the magenta module. (**B**) KEGG analysis on the magenta module. (**C**) GO pathway analysis on the midnightblue module. (**D**) KEGG analysis on the midnightblue module. (**E**) GO analysis on the red module. (**F**) KEGG analysis on the red module. The size of the dots indicates the number of counts, while the color indicates the p-value of each pathway

To find the cellular functions and pathways that are mainly enriched in the key modules, GO and KEGG analyses were performed on the abovementioned midnightblue module, magenta module and red module based on the upstream and downstream relationships of the signaling pathways in the GO functional enrichment and KEGG databases. GO analysis in the magenta module (Fig. 4A) biological processes mainly include binding and release of calcium ions and their corresponding regulatory processes, skeletal muscle development, adaptation, homeostasis, contraction, and systemic processes, and its KEGG pathway analysis (Fig. 4B) includes hypertrophic cardiomyopathy, dilated cardiomyopathy, arrhythmogenic right ventricular cardiomyopathy, cGMP-PKG pathway, cAMP pathway, calcium pathway, etc. The GO analysis (Fig. 4C) in the midnight-blue module of biological processes mainly includes ribosome genesis, processing, metabolism and RNA maturation, and copper ion detoxification and stress response. In contrast, its KEGG pathway analysis (Fig. 4D) includes ribosomal genesis, protein release, pentose phosphate pathway, glutathione metabolism, mineral metabolism, carbon metabolism, and iron death. The GO analysis (Fig. 4E) in the red module biological process mainly includes contraction, development, differentiation and system progress of muscle cell, muscle tissue and skeletal muscle. The KEGG pathway analysis (Fig. 4F) includes cytoskeleton in muscle cells, hypertrophic cardiomyopathy, dilated cardiomyopathy, cGMP-PKG pathway, and AMPK pathway, which is very similar to the KEGG result of the magenta module. Based on these results, it can be assumed that these enriched pathways are mainly involved in muscle, energy substance metabolism, and cell death.

### Screening hub gene

**Fig. 5:**
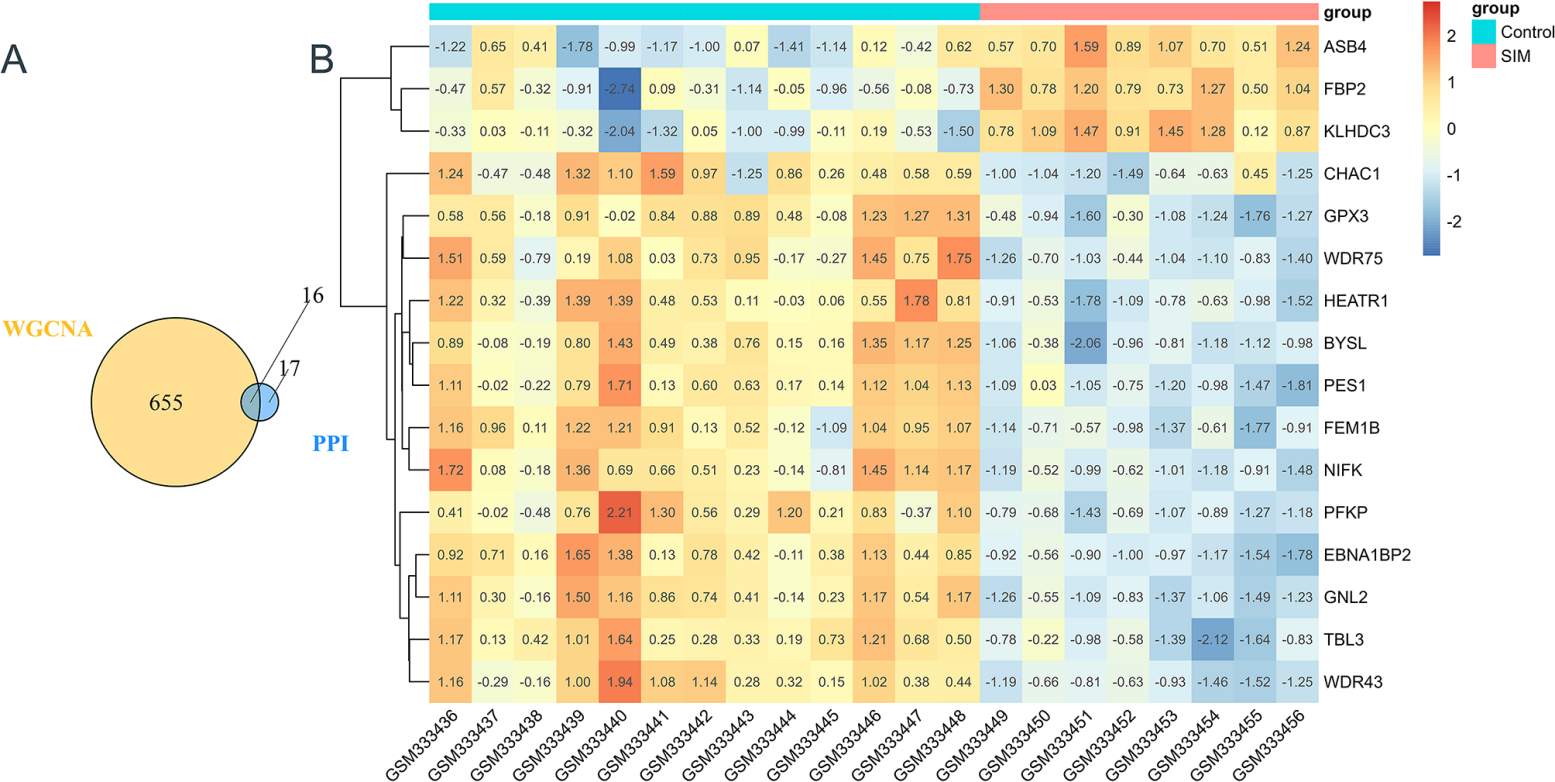
**A**-**B** Hub gene screening. (**A**) Venn diagram for the screening process. (**B**) heatmap shows the different expressions of screened hub genes. Red indicates high expression of genes positively correlated with SIM, and blue indicates high expression of genes negatively correlated with SIM. The numbers in the rectangle represent the normalized gene expression in that sample

WGCNA obtained the three key modules most related to sepsis muscle atrophy, and the genes with GS>0.2 and MM>0.8 were selected from these modules as the candidate hub genes. The Venn diagram was plotted using the R package “VennDiagram.” The intersection of the candidate hub genes screened by the PPI network and WGCNA was obtained (Fig. 5A). The genes in the final overlapping section included FEM1B, KLHDC3, ASB4, GPX3, NIFK, GNL2, EBNA1BP2, PES1, FBP2, PFKP, BYSL, HEATR1, WDR75, TBL3, CHAC1, and WDR43 were selected as the hub genes, which had the strongest positive or negative correlations with septic muscle atrophy. Specifically, KLHDC3, ASB4 and FBP2 were up-regulated. At the same time, FEM1B, GPX3, NIFK, GNL2, EBNA1BP2, PES1, PFKP, BYSL, HEATR1, WDR75, TBL3, CHAC1, and WDR43 were down-regulated (Fig. 5B). The function of these hub genes in the etiology and pathogenesis of septic muscular dystrophy needs to be further investigated. A summary table of all genes in the three key modules and their associated information can be found in supplementary tables.

### The specific roles of our genes of interest were explored by GSEA

**Fig. 6:**
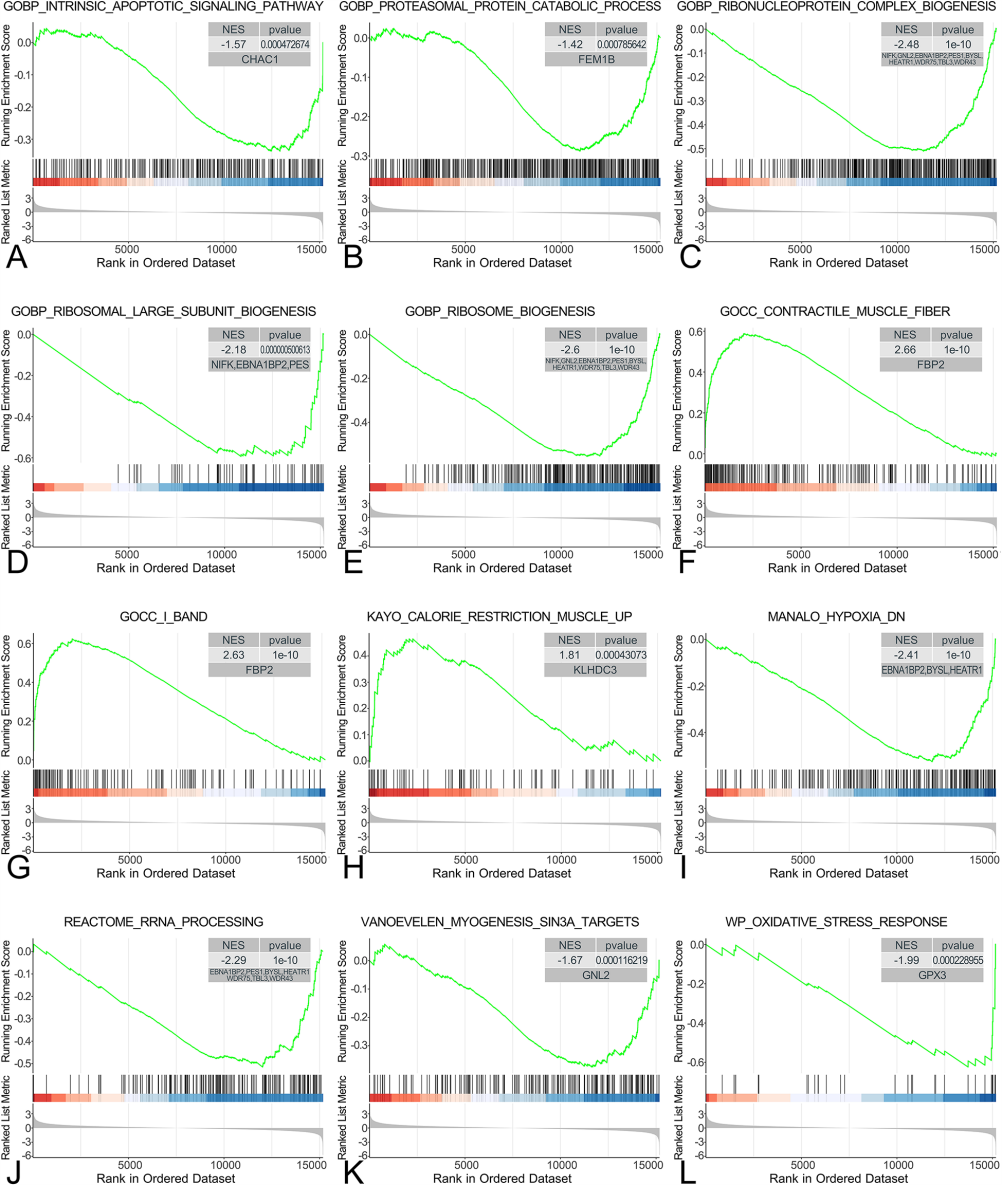
**A**-**L** Gene set enrichment analysis (GSEA). The pathways related to hub genes (**A**) GOBP_INTRINSIC_APOPTOTIC_SIGNALING_PATHWAY, (**B**) GOBP_PROTEASOMAL_PROTEIN_CATABOLIC_PROCESS, (**C**) GOBP_RIBONUCLEOPROTEIN_COMPLEX_BIOGENESIS, (**D**) GOBP_RIBOSOMAL_LARGE_SUBUNIT_BIOGENESIS, (**E**) GOBP_RIBOSOME_BIOGENESIS, (**F**) GOCC_CONTRACTILE_MUSCLE_FIBER, (**G**) GOCC_I_BAND, (**H**) KAYO_CALORIE_RESTRICTION_MUSCLE_UP, (**I**) MANALO_HYPOXIA_DN, (**J**) REACTOME_RRNA_PROCESSING, (**K**) VANOEVELEN_MYOGENESIS_SIN3A_TARGETS, (**L**) WP_OXIDATIVE_STRESS_RESPONSE. The p-values, normalized enrichment scores, and corresponding hub gene names are marked in the upper right corner of each GSEA enrichment plot

The GSEA results show that skeletal muscle structural, oxidative stress response, muscle ganglion function, hypoxia-inducible factors, and rRNA processing modifications are closely related to these 16 hub genes (Fig. 6A-L). These factors are closely associated with skeletal muscle dysfunction, necrosis, and apoptosis of skeletal muscle cells. Therefore, these 16 core genes are considered to be correlated with the presence of SIM. A summary table of GSEA enrichment results for hub genes can be found in the supplementary table.

### Accuracy of hub genes as diagnostic genes

**Fig. 7:**
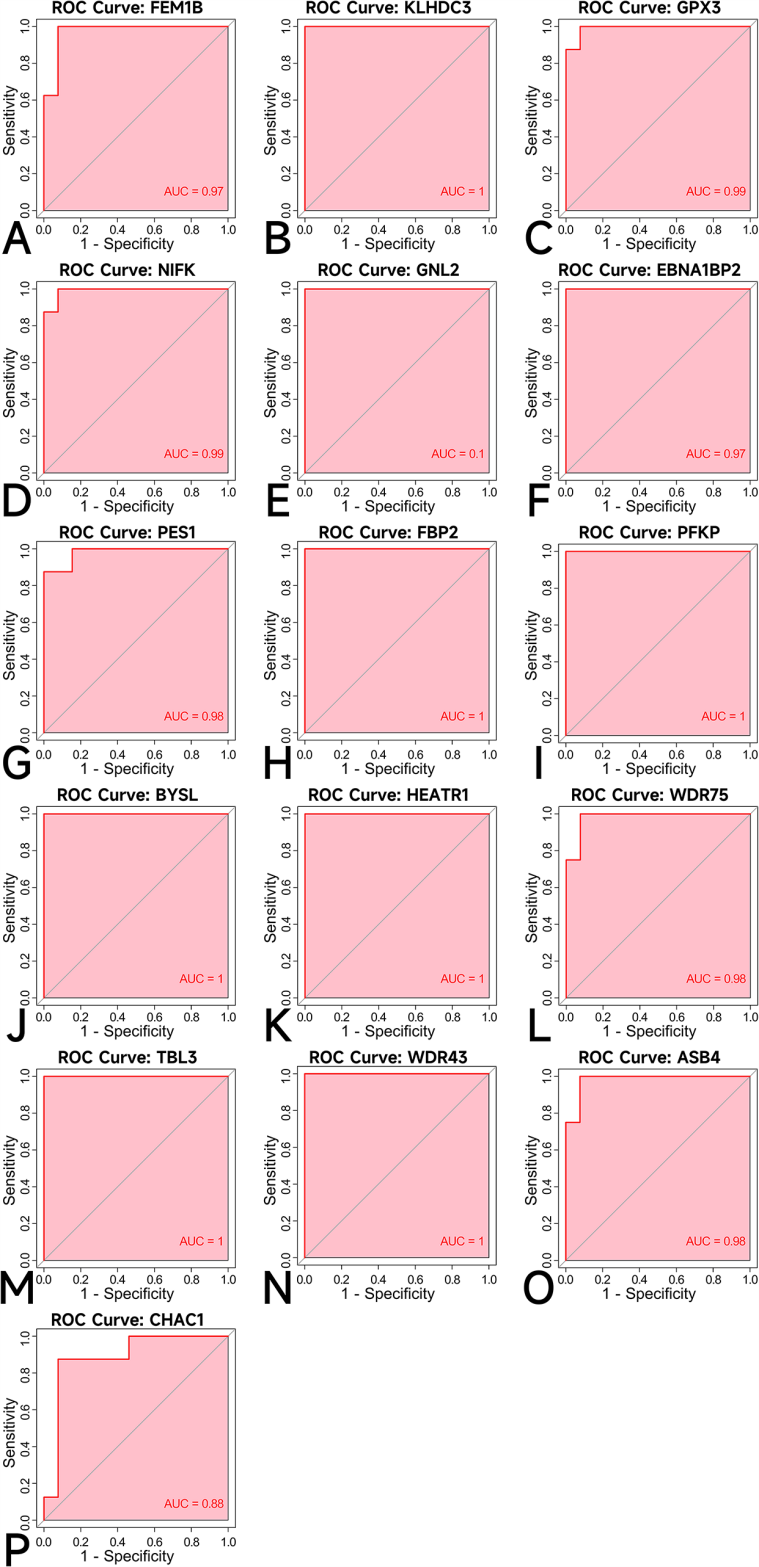
**A**-**P** Quantification of ROC curves. Values of AUC for (**A**) FEM1B, (**B**) KLHDC3, (**C**) GPX3, (**D**) NIFK, (**E**) GNL2, (**F**) EBNA1BP2, (**G**) PES1, (**H**) FBP2, (**I**) PFKP, (**J**) BYSL, (**K**) HEATR1, (**L**) WDR75, (**M**) TBL3, (**N**) WDR43, (**O**) ASB4, (**P**) CHAC1

For each of the obtained pivotal genes, the quantitative calculation of AUC was performed separately as an evaluation index of diagnostic ability (Fig. 7A-P). The AUC of the KLHDC3 gene (Fig. 7B) and FBP2 gene (Fig. 7H) are both 1, the AUC of the ASB4 gene (Fig. 7O) is 0.88, and they have highly expressed genes in SIM (Fig. 5B).

## Discussion

To further explore the genes and mechanisms involved in the pathogenesis of SIM using protein interaction network and weighted co-expression network approaches to screen its related hub genes. Data mining and bioinformatics analysis identified 16 hub genes and several potential biological processes relatively closely related to SIM.

Among the 13 modules obtained, the magenta module and the midnightblue module are the most representative modules connected with SIM. The magenta and the red module are negatively correlated with SIM, while the midnightblue module is positively correlated with SIM. For the midnightblue module GO analysis and KEGG analysis were mainly related to ribosomes, energy substance metabolism, and cell death. The GO analysis in the magenta module is primarily associated with calcium ions and skeletal muscle development, adaptation, homeostasis, contraction, and systemic processes, while its KEGG pathway suggests that SIM is strongly associated with hypertrophic cardiomyopathy, dilated cardiomyopathy, arrhythmogenic right ventricular cardiomyopathy, the cGMP-PKG pathway, the cAMP pathway, and the calcium ion pathway. This is similar to previous findings that energy substances are necessary to maintain intracellular homeostasis. The intracellular balance of energy substances is abnormal during sepsis, leading to skeletal muscle cell injury.

A total of 16 hub genes were screened as our candidate genes for biomarker identification, including 3 up-regulated genes and 13 down-regulated genes. KLHDC3, ASB4 and FBP2 were claimed to be upwardly trending during the development of SIM, whereas FEM1B, GPX3, NIFK, GNL2, EBNA1BP2, PES1, PFKP, BYSL, HEATR1, WDR75, TBL3, CHAC1 and WDR43 downtrend.

KLHDC3 (Kelch Domain Containing 3) is a protein-coding gene whose associated pathways include folding protein responses and cellular responses to stimuli. It has now been reported that the KLHDC3 gene is associated with signaling pathways in skeletal muscle that increase the homeostatic levels of oxidative damage to lipids, DNA, and proteins through the selective up-regulation of transcripts involved in inflammation and oxidative stress and the down-regulation of genes involved in mitochondrial electron transport and oxidative phosphorylation, and thus serve to attenuate the pathological progression of skeletal muscle injury in SIM [27]. Of the 18 genes in the ankyrin repeat sequence and cytokine signaling box inhibitor (ASB) gene family that encode ubiquitin E3 ligases [28], there are 6 skeletal muscle genes (ASB2, ASB4, ASB5, ASB8, ASB12, and ASB1 6) and 3 skeletal muscle/heart genes (ASB10, ASB11, and ASB15). ASB4 is the only one of these nine genes expressed higher in a few non-muscle tissues than in skeletal muscle, namely in the adrenal and pituitary glands [29]. ASB4 is a possible substrate recognition component of the E3 ubiquitin-protein ligase complex, mediating the ubiquitination of target proteins and subsequent proteasomal degradation. Previous publications have not described the cellular role of its ubiquitin ligase activity in striated muscle. Upregulation of the ABS4 gene in sepsis leads to increased skeletal muscle protein loss, which may further lead to the development of SIM. The FBP enzyme is a key enzyme in gluconeogenesis and catalyzes the reverse reaction of fructose-1,6-bisphosphatase [30]. Three distinct groups of FBPase have been identified in eukaryotes and bacteria (FBPase1-3). FBP1 is found in the liver and kidneys, and FBP2 is found in human muscles [31]. FBP2 is a gluconeogenic enzyme that controls the cycle between fructose-6-phosphate and fructose-1,6-bisphosphate, which is essential for regulating whole-body metabolism and thermostability. Induction of the gluconeogenic enzyme FBP2 occurs during exercise training and cold challenges [32], and in these cases, induction of ATP cycling and thermogenesis has also been reported [33]. Recently, it has been shown that overexpression of FBP2 in muscle is sufficient to increase muscle glucose metabolism [34]. However, the current mechanism, the means by which FBP2 causes SIM in sepsis is unclear and requires further research.

Of the remaining 13 down-regulated genes, the GPX3 gene encodes a protein belonging to the glutathione peroxidase family, whose members catalyze the reduction of organic hydroperoxides and hydrogen peroxide (H2O2) by glutathione, thereby protecting cells from oxidative damage [35]. During sepsis, oxidative stress (OS) plays a role in facilitating adaptive responses to hypoxia, bacterial clearance, and endothelial repairing after injury. However, sepsis can cause an imbalance between reactive oxygen species (ROS), reactive nitrogen species (RNS), and the body’s antioxidant system, which can exacerbate multiple aspects of endothelial dysfunction in addition to sepsis-induced mitochondrial dysfunction in a vicious cycle. ROS plays an important role in sepsis-induced endothelial lesions by causing impaired vasodilatation, enhancing leukocyte and platelet adhesion to vascular endothelial cells and capillary permeability, and promoting cell death and a procoagulant state in endothelial cells. At the same time, oxidative stress alters multiple vascular endothelial functions and promotes pro-inflammatory, pro-coagulant, and pro-adhesion phenotypes [36]. Oxidative stress also induces deterioration of the glycocalyx, cell death, increased permeability, and impaired vasoactivity [37]. The biological activity of GPx-3 has been reported to be negatively correlated with mortality and disease severity in patients with sepsis [38]. Thus, the expression of GPX3-related antioxidant pathways is down-regulated during sepsis, thereby exacerbating oxidative stress and possibly contributing to further exacerbation of SIM. GNL2 has been reported in previous studies to be associated with a pathway involved in regulating skeletal muscle ganglion function through modulation of the SIN3A binding site in myotubes [39]. Thus, down-regulated GNL2 is involved in structural defects of skeletal muscle sarcomeres in SIM, leading to the deterioration of SIM. HEATR1 and BYSL have been reported to be down-regulated in association with overexpression of HIF-1, which undergoes dynamic changes in expression during sepsis, regulating inflammatory responses in the acute phase and protective responses in the late phase and has a significant impact on cytokine production, metabolism, cellular adaptation, and clinical symptoms [40]. So, the role of the HEATR1 gene in SIM pathophysiology may also be time-dependent.WDR43 has been reported to be associated with a fibroblast pathway that can express the sustainably active Rho (Q63L) family [41]. Rho family proteins are identified as key regulators of endothelial barrier function by modulating endothelial permeability [42]. RhoA stimulates actin-cytoskeleton reorganization and cell contraction through its downstream effector Rho kinase (ROCK), leading to connexin remodeling and loss of endothelial barrier integrity [43]. Thus, aberrant RhoA/ROCK signaling cascade stimulation is a mechanism of endothelial barrier dysfunction in sepsis. Whereas, unlike endothelial cells, in vascular smooth muscle cells, the RhoA/ROCK pathway mediates the contractile response as a calcium-sensitizing mechanism. Most studies have demonstrated inhibition of this system, since a reduction in the activity of the Rho component due to the low expression of WDR43, with a consequent reduction in the inhibitory effect of ROCK on myosin phosphatase, leading to vasodilatation, vasotruncation, and hypotension in models of sepsis status [44]. Therefore, low expression of WDR43 in SIM may play a role in attenuating the Rho pathway and subsequently protecting the vascular endothelium and vascular tone in sepsis, thereby slowing down skeletal muscle injury in SIM. EBNA1-binding protein 2 (EBNA1BP2) is a protein-coding gene. Pescadillo ribosome biogenesis factor 1 (PES1) is also a protein-coding gene. Pathways related to both EBNA1BP2 and PES1 include rRNA processing in the nucleus and cytoplasmic lysosomes, as well as intron-containing pre-mRNA processing [45, 46]. The results of EBNA1BP2 and PES1 analyzed in GSEA showed that the low expression of both related gene sets was associated with B lymphocytes. The low expression of EBNA1BP2 was reported to be associated with CD5-positive B lymphocytes [47]. The set of PES1-related genes is involved in the regulatory network to reconstitute B lymphocyte expression profiles. In contrast, the differential activity of the network connecting the co-regulated genes determines the B lymphocyte phenotype [48]. In patients with sepsis, a reduced number of circulating B cells usually portends a poor prognosis [49]. Different B cell subpopulations are not uniformly affected in patients with sepsis. In contrast, selective reductions in circulating B cell subpopulations can be attributed to endogenous and exogenous apoptosis as well as cellular pyropoiesis [50], and their generalized depletion and impaired maturation are associated with it [51]. Thus, it can be currently considered that EBNA1BP2 and PES1 may be involved in the pathological process of sepsis and SIM by regulating the number and function of B lymphocytes. However, the current roles and mechanisms of different subpopulations of B cells in SIM are still unclear and need further investigation. WDR75 is a nucleolin protein involved in pre-rRNA processing and regulation of rRNA transcription. Current knowledge of WDR75 is still very limited, and studies on its association with disease are limited to a few cancers. Uhlen et al. analyzed the pathological features of the human cancer transcriptome and found that abnormally high expression of WDR75 mRNA was a poor prognostic factor for renal and hepatocellular carcinomas [52]. In a recent study, researchers knocked down WDR75 in various cultured human cell models. In both tumor and non-tumor cell models, this process activated the RPL5/RPL11-dependent stabilization of the p53 checkpoint, ultimately leading to impaired cell proliferation and senescence [53]. Transducin beta-like 3 (TBL3) is also a protein-coding gene with the same intracellular pathway associated with WDR75. The current mechanism of action of WDR75 and TBL3 in patients with sepsis is unclear, and further studies are needed. FEM1B encodes an anchor protein repeat protein that belongs to the death receptor-related protein family and plays a role in mediating apoptosis [54]. The protein encoded by NIFK can bind RNA and may play a role in mitosis and cell cycle processes [55]. Meanwhile, the pathways enriched by GSEA that are related to both are proteasomal protein catabolic process, ribosomal large subunit biogenesis. The pathway involved in FEM1B is the chemical reaction and pathway in which peptidyl bond hydrolysis mediated by the proteasome leads to the breakdown. The pathway that NIFK is involved in leads to the biosynthesis, assembly, and arrangement of the large ribosomal subunit components of the cell process, including transport to the protein synthesis site. The mechanism by which FEM1B and NIFK lead to SIM in septic patients is unclear and requires further research. The enzyme encoded by PFKP is the platelet-specific isoform of the fructose-6-phosphate kinase subtype, which plays a key role in regulating glycolysis. PFKP regulates cell proliferation, apoptosis, autophagy, cell migration/migration, and stemness through glycolytic and non-glycolytic non-dependent functions. PFKP plays a role in the cytoplasm and the cell membrane, mitochondria, lysosomal membrane, and nucleus [56]. Currently, no research has been found on the correlation between PFKP and sepsis and SIM. The specific mechanism of its upregulation leading to SIM requires further research. ChaC Glutathione Specific Gamma-Glutamylcyclotransferase 1 (CHAC1) is a protein-coding gene. Its related pathways include cellular response to stimuli and metabolic pathway biotransformation I and II. Recently, ChaC glutathione-specific γ-glutamylcyclotransferase (CHAC), including CHAC1 and CHAC2, has been identified as an intracellular glutathione degrading enzyme [57], resulting in depletion of intracellular glutathione under stress conditions. While human CHAC2 expression is ubiquitous, CHAC1 expression is highly enriched in skeletal muscle. CHAC1 is universally upregulated in various muscle-wasting conditions [58]. CHAC1 inactivation preserves glutathione in skeletal muscle under both physiological and pathological conditions. In addition to being a glutathione degrading enzyme, CHAC1 may have other functions. The literature reports that CHAC1 is an apoptosis-promoting gene involved in the unfolded protein response regulated by the ATF4-ATF3-CHOP pathway [59, 60]. However, in contrast, in our study, there was a correlation between skeletal muscle atrophy and the downregulation of CHAC1 in most SIM patients. The researchers consider this related to sepsis, and the specific mechanism still needs further research.

This study needs to take into account that there are still some limitations. The sample size in this study was small, and studies with more significant amounts of data are required in order to validate our results. Many other factors influence sepsis-complicated skeletal muscle injury or atrophy. In the follow-up study, considering that obtaining skeletal muscle samples from sepsis patients in the clinical environment is challenging, gene expression levels can be verified through animal or cell experiments in vitro. Secondly, the molecular function mechanism in SIM cell phenotype or animal phenotype can be detected by overexpression or inhibition of target genes. While the area under the ROC curve can represent the accuracy of a molecule as a diagnostic biomarker, additional measures in the clinic are required to confirm its specificity and sensitivity.

## Conclusion

We screened 16 promising SIM biomarker genes (KLHDC3, ASB4, FBP2, FEM1B, GPX3, NIFK, GNL2, EBNA1BP2, PES1, PFKP, BYSL, HEATR1, WDR75, TBL3, CHAC1, WDR43) of SIM by PPI and WGCNA methods, and also detected the expression of HIF, Rho and CD5-positive B cell-related genes are closely associated with SIM. Further experiments are needed to validate the related hub genes and their roles in SIM.

## Electronic supplementary material

Below is the link to the electronic supplementary material.

Supplementary Material 1

Supplementary Material 2

## Data Availability

The dataset GSE13205 analyzed during the current study is available in the GEO repository (https://www.ncbi.nlm.nih.gov/geo/query/acc.cgiacc=GSE13205) for analysis.

## Abbreviations

SIM: Sepsis-induced myopathy
WGCNA: Weighted gene co-expression network analysis
PPI: Protein-protein interaction
STRING: The search tool for the retrieval of interacting genes
MCODE: Molecular complex detection
TOM: Topological overlap matrix
GS: Gene significance
MS: Module significance
ME: Module eigengene
GO: Gene ontology
KEGG: Kyoto encyclopedia of genes and genomes
MM: Module membership
GSEA: Gene set enrichment analysis
NOM p-val: Nominal p-value
NES: Normalized enrichment score
ROC: Receiver operating characteristic
AUC: Area under the curve
KLHDC3: Kelch domain containing 3
H2O2: Hydrogen peroxide
OS: Oxidative stress
ROS: Reactive oxygen species
RNS: Reactive nitrogen species
ROCK: Rho kinase
EBNA1BP2: EBNA1-binding protein 2
PES1: Pescadillo ribosome biogenesis factor 1
TBL3: Transducin beta-like 3
